# What Is Fittest in Homœopathy and Likely to Survive

**Published:** 1893-07

**Authors:** 


### HOMŒOPATHIC PHYSICIAN,

A MONTHLY JOURNAL OF

HOMŒOPATHIC MATERIA MEDICA AND CLINICAL MEDICINE.

"If our school ever gives up the strict inductive method of Hahnemann, we are lost, and deserve only to be mentioned as a caricature in the history of medicine."—CONSTANTINE HERING.

Vol. XIII. JULY, 1893. No. 7.

### EDITORIAL.

WHAT IS FITTEST IN HOMŒOPATHY AND LIKELY TO SURVIVE.—In the *North American Journal of Homœopathy* for July is an address bearing the above title, by Dr. W. S. Searle, of Brooklyn.

The author takes a retrospect of the three-quarters of a century during which Homœopathy has been "on trial" before the world, and assumes that during that time Homœopathy has been tested and investigated by the great body of physicians who have professed to practice its principles, and that "whatever is true in Homœopathy, whatever has stood the test of modern scientific thought and experiment is gradually being accepted by the medical profession in general. And what is fanciful and false has been, or is being, rejected by the homœopathic school itself."

Looking in the direction that Dr. Searle points, and considering with him this same "no small portion of the history of medicine," it is a little difficult to perceive that the great body of the advocates of the law of the similars have made any serious investigation of the merits of that law or have in any way been instrumental in separating the truth from the error.

There are several classes of men who have come into the school and assumed to practice its tenets, but the results of the work of the majority of them are anything but satisfactory, anything but conclusive to those who wish to know what truth there be in the method of Hahnemann.

One class, and a very large one, came into the homœopathic school, not because they were convinced that there was something radically wrong in the treatment of the dominant school; that Homœopathy represented a reform in medical practice; that it meant a genuine and momentous discovery for the good of mankind. They came because they were attracted by the pecuniary success of the old masters in Homœopathy, who made themselves celebrated by their marvelous homœopathic cures, brought Homœopathy forward and compelled the attention of the medical world to its claims, and incidentally, therefore, created a *trade name* of high commercial value and with the added advantage of less competition. It was this *trade name* that this class of men embraced, of course with the hope of pecuniary gain, not the distinctive principles themselves. As their minds had not previously been prepared for the principles, they were not likely to accept them, when by assuming the name of homœopathic practitioners, they were confronted with them. On the contrary, they did not hesitate to deny them and to combat them with as much virulence as the bitterest enemies of the reigning school, and openly to practice old-school methods which they asserted were homœopathic. Hence we have the remarkable phenomenon of a set of doctors vigorously denying and denouncing the principles they professed to believe were the best adapted to relieve the distresses of disease, to the scandal of the laity, and to the merriment of our enemies in the old school, who did not hesitate to use it as an argument against Homœopathy in general.

It cannot be possible that the judgment of *this* class shall be taken as to what is true or false in Homœopathy.

Another class whose numbers contribute to make up the great majority who nominally practice Homœopathy are the graduates of homœopathic colleges. However well prepared they may be upon the ologies, specialties, and general therapeutic measures of the old school freely taught as a part of a "liberal medical education" by these colleges, they have yet but scant training in the "method" of Homœopathy. Some of these men have not even heard of *The Organon*, or they know it only in a vague sort of way without any comprehension of its deep significance. They have, in fact, been *deprived* of it during their student life, there having been a settled policy on the part of many professors to avoid giving any instruction in its pages. This policy has been defended by quoting the well-known saying: "*The Organon* should be read not perhaps by the student, but by the educated earnest practitioner." A remark so hypocritical, so crafty, at once excites surprise and invites inquiry into the motives of the originator. It certainly justifies the belief that the intention was to prevent graduates from making any attempt to put homœopathic principles into actual use. The practical result of it has been, as just stated, to organize an army of doctors whose ideas of Homœopathy were quite vague and who will be found to harbor a latent hostility to it.

It cannot be possible that the judgment of *this* class shall be taken as to what is true or false in Homœopathy.

Another class to be considered. They are average practitioners, graduates of homœopathic colleges, with little confidence in themselves, who, whenever they meet with a case which is difficult, at once call in the aid of some one of the professors of their *Alma Mater*. It has been alleged of one or two colleges that the professors will not permit the education of the students to pass a certain point, as it is desired to keep them in a state of dependence upon the *Alma Mater* that, by the frequent consultations with the professors, an increase of practice and prestige may be enjoyed by the latter, and of course an increase of income. However this may be, there is such a class, and it helps to swell the majority assuming to practice Homœopathy.

It cannot be possible that the judgment of *this* class shall be taken as to what is true or false in Homœopathy.

Finally, we have the class of the distinguished men of the school. Men who have, by their energy, their industry, the tenacity of their opinions, the boldness and aggressiveness with which they have asserted these opinions, and the brilliancy of their writings, become advanced to the front ranks of the profession. Men who are looked up to by the great majority as leaders in the thought of the school. Men who are considered as authorities, and have in consequence crystallized that thought into the views referred to by Dr. Searle as the accepted sentiment of the school.

Yet this class is responsible for the state of opinion prevailing in the classes previously considered. Thus, one distinguished veteran of this class, in a celebrated article written some years ago, and now freely quoted by Dr. Searle, gives a series of conditions in which Homœopathy is of no avail in therapeutics. By the method of exclusion he finds that Homœopathy is of no use in this condition and that, and so he relegates the law of the similars to an obscure corner by which it is rendered inoperative in all but a very few cases. By thus limiting the field of its usefulness, he opens wide the entrance to the practices of the old school which homœopathists are understood to have abandoned for a more enlightened method.

Another distinguished writer has classed Hahnemann's writings under two great heads: the *certis* and the *dubiis*.

The *certis* consists of the formula *Similia Similibus Curantur*, which, according to this writer, is not a law, but a good rule which may or may not be used at the practitioner's convenience or caprice. The *dubiis* includes everything else that Hahnemann wrote. Here again Homœopathy is crowded into a corner and the door opened wide for the use of old-school methods under the homœopathic name. Both these writings are indeed nothing else than rational pleas to justify these departures from homœopathic "methods." Both writings have been seized upon by acute and implacable enemies in the old school for sharp and derisive articles against Homœopathy and impugning the sincerity of its advocates.

The most clever essays against Homœopathy have been written by physicians of the regular school who have been bright enough to scan the literature of our school for articles like the two just referred to, and then to quote them against us. Such arguments are all but unanswerable, and have a large influence in hindering the development of the school, in discouraging any inquiring mind from investigating the truth in the new-school principles, because our own disciples practically tell them "there's nothing in it."

These homœopathic authors, writing in this objectionable way, have simply followed the methods of the philosophers of the past age, who depended for their scientific views upon *à priori* reasoning, instead of the inductive results of experiment.

Looking backward over the history of physical science, the distinguishing difference between the philosophers antedating Sir Isaac Newton on the one hand, and that distinguished man and those who followed him on the other, was this very thing of the use of experiment to get a correct scientific horizon and the avoidance of pure reasoning, which, in the absence of experiment, was bound to lead to error. In short, it was the introduction of the *inductive method* that sent physical science forward with such giant strides. The only class of thinkers who lagged behind and failed to avail themselves of the inductive method was the medical profession. Suddenly Hahnemann stepped out from among them and personally pursued the experimental method after the manner of the physicists, and contemporaneously, too, with a group of the most distinguished of them who made memorable the first quarter of the present century. Hahnemann's reward was the same as theirs—a grand discovery.

The distinguished authors of whom we complain do not appear to have adopted this experimental mode. Like the ancient philosophers, they seem to have resorted to pure reasoning, with the result of finding Hahnemann's teachings for all practical purposes a fallacy.

Now, how can the judgment of the gentlemen of this class, who, notwithstanding their attainments have made such signal mistakes, be accepted as to what is true or false in Homœopathy?

The merits of the majority professing to practice Homœopathy being thus considered, what of the minority? Who are they? A small band of devoted followers of Hahnemann, stigmatized variously as "enthusiasts," "transcendentalists," "Hahnemanniacs," etc., who faithfully apply the Hahnemannian principles to every case that comes under their notice; strictly follow the teachings of *The Organon,* carefully watch the results and record them, and when baffled by failure seek the error which caused the failure. The testimony so accumulated will sooner or later form a mass of evidence that will establish the truth of Homœopathy or prove it an error. This step-by-step process is the only way in which the truth will be established. It is the way in which the truths of the collateral sciences have been established and their errors eliminated. Without it Homœopathy will never progress, doubt of there being any truth at all in it will continue to hang over it, scientific men will refuse to investigate it, and humanity will continue to endure much suffering which could be annihilated.

Whilst Homœopathy is enduring this hindrance, the dominant school is gradually approaching the homœopathic point of view; not, as intimated by Dr. Searle, through adoption of the views of the majority in our school, for they scornfully reject the teachings to which we have alluded, and make fun of them, but by the slow process of evolution. It would not be too extravagant to expect to behold the curious phenomenon of the two schools exchanging places, the old school becoming ardent homœopathists, the new school taking the rational ground of the allopathic school.

